# Experimental selection of rolling contact length to the average thickness (L\h_ave_) ratio for producing fine-grain Incoloy 907 superalloy sheet by hot-rolling

**DOI:** 10.1016/j.heliyon.2024.e34335

**Published:** 2024-07-09

**Authors:** Seyed Sajjad Babaei Sangtabi, Seyed Mehdi Abbasi, Rashid Mahdavi

**Affiliations:** Faculty of Materials & Manufacturing Technologies, Malek Ashtar University of Technology, Tehran, Iran

**Keywords:** Incoloy 907, Hot-rolling, Thickness reductions, Banded structure, Ductility

## Abstract

The current study investigates the effects of L\h_ave_ values on the microstructure and tensile properties of the superalloy Incoloy 907. These effects are studied using hot-rolling at 84 % and 96 % thickness reductions. Microstructural examination of the received sample revealed a non-uniform coarse grain structure with precipitates of oxide phases rich in niobium and Laves. These precipitates are shredded and randomly distributed in the microstructure with elongated grains after hot-rolling under 84 % reduction. Following the solution-treatment, this sample's simultaneous static recrystallization and precipitation of secondary-phase particles has formed a banded structure of grain-size and secondary-phase particle bands. The particles of the secondary-phases are locally accumulated on the material's surface in the 96 % hot-rolled sample, creating a duplex grain and non-random microstructures with different cross-sectional conditions. The secondary-phase particle bands caused by the previous solution-treatment are retained in the pre-hot-rolled sample under 84 % reduction after aging. Furthermore, XRD analysis results show that the precipitation peak of γ'/γ phase was more visible and intense in this sample. Tensile tests conducted at room temperature and 649 °C indicate that the pre-hot-rolled sample with an 84 % reduction exhibited a 1 % improvement in cold ductility and an 8 % enhancement in hot ductility compared to the sample with a 96 % reduction.

## Highlights

• Investigating the rolling contact length to the average thickness (L\h_ave_) ratio for producing Incoloy 907 superalloy sheet using hot rolling.
• Formation of banded structure of grain-size and secondary-phase particle bands in Incoloy 907 superalloy sheet.
• Creating a duplex grain and non-random microstructures with different cross-sectional conditions in Incoloy 907 superalloy sheet.
• Choosing an optimal and normalized ratio in hot rolling billet to Incoloy 907 superalloy sheet for direct use in high-temperature structural applications.

## Graphical abstract

**Figure:**
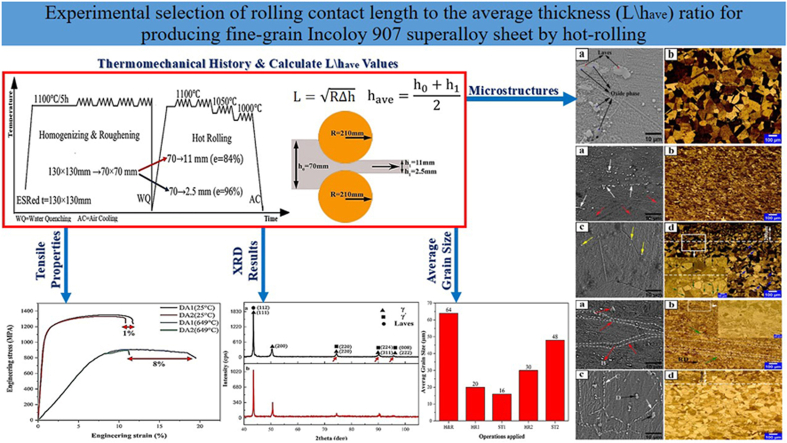

## 1 Introduction

Incoloy 907 superalloy is based on Fe–Ni–Co elements. It is known for its thermal expansion coefficient, which is approximately 40 % lower than conventional superalloys, excellent resistance to hydrogen embrittlement, and high strength up to a maximum temperature of 650 °C. To use Incoloy 907 alloy in high-temperature structures, Nb or Ti is added to the solid solution of Fe–Ni–Co to improve the precipitation hardening of the γ′ phase. In addition to the phases that occur during aging, carbon in 900-series alloys promotes the solid formation of Nb-rich MC (cubic) carbides during the thermomechanical process and heat treatment, which are typically aligned with the rolling direction. Laves and silicon-rich G phases were provably stable at solution temperatures above 980 °C in phase transformation studies [[1], [2], [3]]. As with the ferrite, perlite, and oxide phases, the Laves phase in Incoloy 909 and Incoloy 907 superalloys can appear as bands in steels [4,5]. Because of chemical differences between the dendritic nucleus and the inter-dendritic regions, bands often form during unstabled freezing. Furthermore, delamination primarily depends on three factors: (1) the alloying elements' microsegregation, (2) the cooling rate (or, more broadly, the imposed temperature profile) during the transformation, and (3) the austenite grain size. Previous studies [6,7] used electron probe microanalysis (EPMA) to show that ferrite/perlite bands in wrought steel were strongly associated with alloying elements' microsegregation. Normalizing high temperatures can sometimes remove a steel's delamination. If this steel is hot-rolled (or deformed) and then austenitized again, the delamination will increase gradually. This increase is due to an increase in deformation. Aside from delamination, heterogeneity of deformation along the thickness of the sheet can cause surface defects when hot-rolling low-thickness superalloy sheets with higher friction. A sticky friction zone in the material greatly increases the rolling force. Moreover, the shear stress caused by friction is proportional to the contact length to the material's average thickness ratio. In other words, as this ratio increases, so does the shear stress caused by friction. It is worth noting that this ratio increase corresponds to an increase in the radius of the roller, a more significant decrease in thickness, or a decrease in the material's initial thickness. Therefore, the contact length ratio to the material's average thickness should be as small as possible to reduce the shear stress and the rolling force. The excessive reduction in the contact length ratio to average thickness means that the deformation is concentrated and localized, increasing the extra work and deformation power. The optimal and normalized value of the contact length to the average thickness of the material is usually different for each material and thickness, and it can be obtained in such a way that the rolling force and power have the lowest possible value. This issue is exacerbated by hot-rolling processes, which have a much higher friction coefficient than cold rolling [[8], [9], [10], [11], [12]]. In general, superalloys' microstructure and desired final properties are primarily determined by the control of thermomechanical process parameters and heat treatment type [13]. Because there have been few published studies [[1], [2], [3], [4], [5]] on thermomechanical processes on Incoloy 907 superalloy, and because 900 series superalloys are sensitive to duplex grain structure [14], it is critical to investigate the effect of applied strain during hot-rolling on the microstructure and mechanical properties of Incoloy 907 superalloy. Also, converting ingots to sheets, common in large-scale manufacturing, is a complex and expensive process for superalloys. Therefore, in this study, the effects of L\h_ave_ values after hot-rolling and the subsequent heat treatment (solution and two-stage aging) have been discussed by microstructural studies, ambient temperature, and high-temperature tensile tests to select the optimal and normalized value of L\h_ave_, increase the efficiency of sheet production as a final product, and achieve a suitable combination of tensile properties of ambient and high temperature.

## 2 Experiments

### 2.1 Introducing the material and its production method

This study uses an Incoloy 907 superalloy ingot. The alloy is melted and cast in a vacuum induction melting (VIM) furnace. The alloy is then refined via the Electro-slag Remelting (ESR) process. Table 1 shows the chemical composition analysis by spark emission spectrometer. A 2004 Spectro spark emission spectrometer is used for this analysis. Also, LECO elemental analyzer is used to measure oxygen.1.5 % wt titanium is added to the slag during the ESR process. The ESR slag used contains 70 % CaF_2_ and 30 % Al_2_O_3_.

**Table 1:** Analysis of the chemical composition of the produced alloy.

chemical composition(Wt.%)	Ni	Co	Nb	Ti	Al	Si	O	Fe
UNS N19907 [15]	35–40	12–16	4.3–5.2	1.3–1.8	0.2>	0.07–0.35	20-30 PPM	Remaining
Present analysis	34.3	14.1	5.1	1.5	0.1	0.2	90 PPM	Remaining

**Fig. 1:**
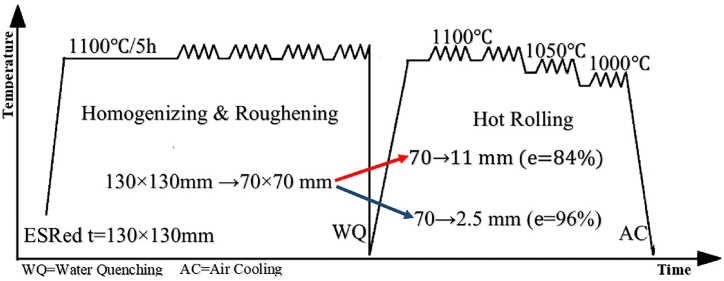
A schematic representation of the thermomechanical history performed on Incoloy 907 superalloy.

The ingot obtained from the ESR process (dimensions 130 mm × 130 mm) was subjected to homogenization and roughing treatments (forging + hot-rolling) until reaching the dimensions of 70 mm × 70 mm for the following reasons: the local separation of Nb elements (potential segregation) during the VIM process, which was not resolved during the ESR process, and the loss of the dendritic structure [13]. Next, the 70 mm thick billet was divided into two parts to convert it into a sheet while simultaneously studying the effect of L\h_ave_. The first part was hot-rolled with an 84 % reduction in successive passes up to 11 mm thickness. The second part was hot-rolled with a 96 % reduction in successive passes up to a thickness of 2.5 mm. The samples were then subjected to similar solution and two-stage aging treatment. Furthermore, a rolling machine with a roller radius of 210 mm and a speed of 6.5 RPM is used for hot-rolling. Fig. 1 and Table 2 show the schematic representation and details of the thermomechanical history and heat treatments performed. The interrupt time between passes in hot-rolling this alloy is less than 1 min. Moreover, during the hot-rolling process, the temperature is gradually decreased and uniformized across the sample volume by subjecting the sample to the furnace for 10 min at various temperatures (1100 °C»1050 °C»1000 °C). Furthermore, in this study, the reduction of hot rolling in all passes is approximately e = 10–11 %. In order to achieve thicknesses of 11 mm and 2.5 mm, 8 passes and 9 passes of hot rolling were conducted, respectively.

**Table 2:** Parameters used for heat treatment of hot-rolled sheets.

Thickness and sample code	Operations applied
70 mm - H&R	Homogenized and Roughened (sample received)
11 mm - HR1	Hot-rolled
11 mm - ST1	Solution-Treated in 980 °C × 1 h, AC
11 mm - DA1	Solution-Treated and Two-Stage Aged in 720 °C × 8 h, FC(55 °C/h)-620 °C × 8 h, AC
2.5 mm- HR2	Hot-rolled
2.5 mm - ST2	Solution Treated in 980 °C × 1 h, AC
2.5 mm - DA2	Solution-Treated and Two-Stage Aged in 720 °C × 8 h, FC(55 °C/h)-620 °C × 8 h, AC

### 2.2 Calculating L\h_ave_ values for the present work

**Fig. 2:**
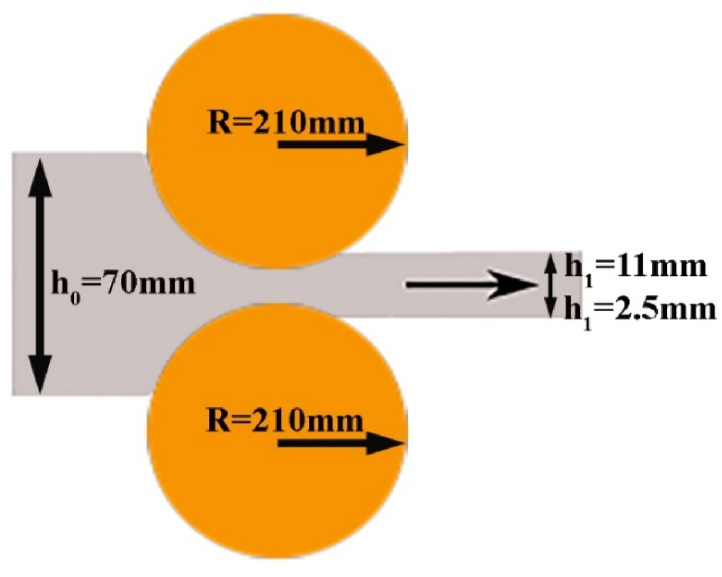
Schematic representation of the parameters used in equations (1), (2)).

For various metals and thicknesses, the optimal and normalized mode L\h_ave_ value varies [[8], [9], [10], [11], [12]]. Equation (1) defines the rolling contact length (L), and Equation (2) defines the average material thickness (h_ave_) [[16], [17], [18]].

(1) $$L=\sqrt{R\Delta h}$$

$$\Delta h=h_{0}-h_{1}$$

(2) $$h_{\text{ave}}=\frac{h_{0}+h_{1}}{2}$$

where R is the roller radius, Δh is the amount of material thickness reduction after one rolling step, h_0_ is the material thickness before rolling, and h_1_ is the material thickness after rolling, as shown schematically in Fig. 2. Accordingly, this ratio is L\h_ave_ = 1.36 for samples with a thickness of 11 mm and L\h_ave_ = 2.87 for samples with a thickness of 2.5 mm.

### 2.3 Microstructure investigation experiments

In the current study, the samples were mechanically cut for each test based on the specified in their standard. An etch solution containing a combination of 16 FeCl_3_ gr, 50 ml HCl, and 1 ml HNO_3_ [19] is used for microstructural analysis. Olympus BX51 optical microscope is employed to observe and study samples’ structure. ImageJ image analysis software and the ASTM E112 standard [20] are used to calculate the grain size. Vega TESCAN electron microscope is also used for further microstructure studies. Furthermore, the energy-dispersive X-ray spectroscopy (EDS) analyzer system investigates the chemical composition of Laves and oxide phases. In this study, all the microscopic images and characteristics are based on the RD-TD plane. Also, the SEM images were taken only from the central areas of this plane (not the areas close to the surface of the sheets). XRD analysis of the BRUKER device is employed to evaluate the phases present in the microstructure, particularly the phases after aging. For this experiment, a copper anode with λ = 1.54 α is used, and the ICOD reference cards with the 0723-005-00, 0908-017-00, and 1417-047-00 codes in Plus Xpert Highscore software are used, respectively, to identify the γ′ and Laves phases and the matrix γ.

### 2.4 Tensile tests at ambient and 649 °C temperatures

Tensile test samples are prepared per E8 [21] and E21 [22] standards for ambient and 649 °C temperatures. For this purpose, tensile samples are selected in the longitudinal rolling direction. The experiment uses a sub-size sample with a 12.5 mm gauge length, 2 mm thickness, and 3 mm width. Tensile testing is performed by Instron 8502 and a strain rate of 0.028 s^−1^ at both temperatures. Also, this test was repeated 3 times for each sample.

## 3 Results and discussion

### 3.1 Investigation of the primary microstructure

**Fig. 3:**
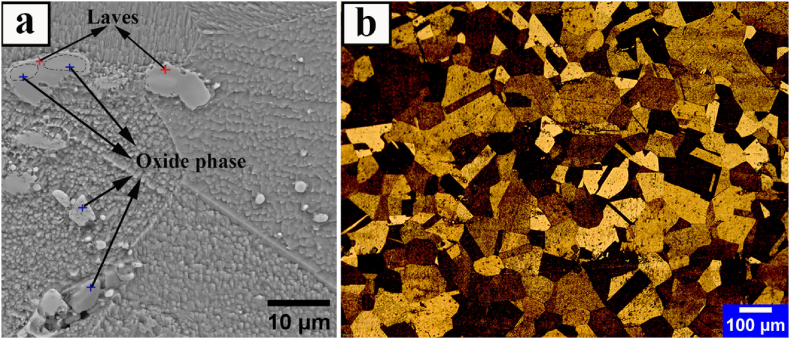
The images of the received roughened sample of Incoloy 907 superalloy with classifying precipitates of secondary-phases; a) SEM b) OM.

Fig. 3 depicts the received microstructure (t = 70 mm) of Incoloy 907 superalloy after homogenization and roughing at a constant temperature of 1100 °C. As shown in Fig. 3, the microstructure consists of a non-uniform coarse grain structure with an average grain size of about 64 μm. The grain growth appears to be complete due to the high temperature of the process and the lack of buckling in the grain boundaries.

Table 3 shows the results of EDS analysis on the precipitates seen in Fig. 3a, indicating that these precipitates are niobium-rich oxide phases. Due to the strong oxidation tendency, adding pure Ti to the ESR process slag reduces the molten oxygen and, as a result, increases the oxidative precipitates [[23], [24], [25]].

**Table 3:** EDS analysis of the particles in the SEM microstructure of Fig. 3.

ELM (Wt%)	Fe	Ni	Co	Nb	Ti	Si	O
Laves	23.9	25.4	15.2	31.2	0.8	3.3	–
Oxid phase	19.2	16.1	9.2	47.8	1.0	1.0	5.7

Also, in a study conducted by researchers [26], it was demonstrated that altering the cooling rate during solidification leads to a significant change in the distribution of oxide particles. As the cooling rate increases, the number of oxides rises while their size decreases. This indicates that the driving force for particle precipitation and growth, known as supersaturation, is influenced by variations in the cooling rate. Moreover, it is established that intermetallic compounds like the Laves phase tend to precipitate both homogeneously and heterogeneously in Fe–Ni–Co-based alloys containing supersaturated elements such as Nb, Si, and Ti when subjected to temperatures ranging from 800 °C to 1040 °C [5,19,27,28]. According to classical nucleation theory, heterogeneous nucleation has a lower energy barrier and critical size compared to homogeneous nucleation, making it a more stable process with lower Gibbs free energy [29,30]. Consequently, the Laves phase preferentially formes heterogeneously around some precipitates at time intervals of quenching in water. Ultimately, it can be concluded that the oxide phases are the nucleus of Laves phase formation. As shown in the previous research [31], the temperature range of the formation and dissolution of the oxide phase is 1175-1100 °C. According to the thermomechanical history shown in Fig. 1, therefore, the oxide phase can form both during solidification and during thermomechanical treatment. Likewise, a similar occurrence has been documented in the precipitation structure of a carbo-nitride within the IN718 superalloy [32]. In this case, an MgO.Al_2_O_3_ core surrounded by TiN served as the nucleation site for a primary MC carbide.

### 3.2 The effect of L/h_ave_ on the microstructure of hot-rolled and solution-treated samples

**Fig. 4:**
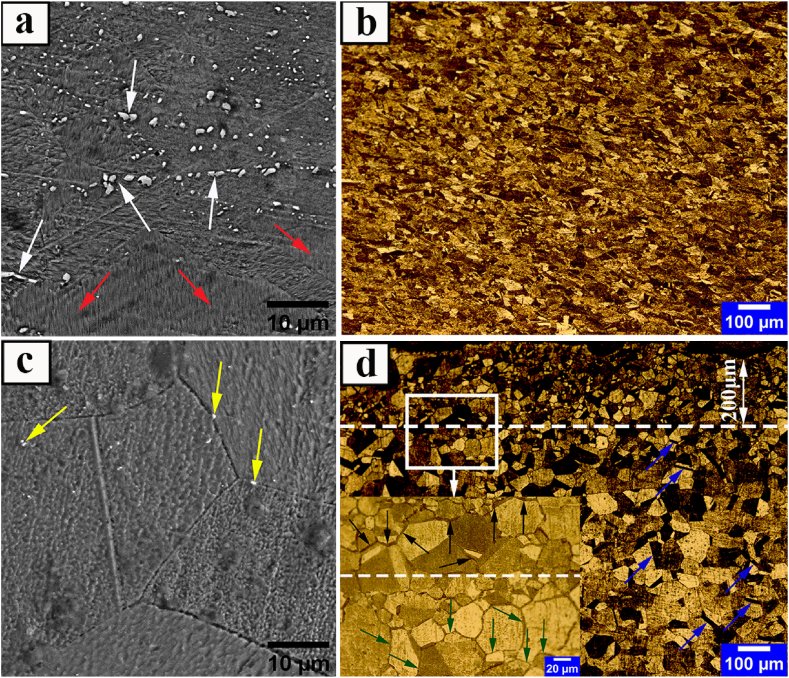
OM and SEM-BSE images of hot-rolled Incoloy 907 superalloy samples; a-b) HR1, c-d) HR2. Shredded secondary-phases in the rolling direction, areas free of secondary-phases, Very fine precipitates, undistorted annealing twins, grain boundaries with less thickness, and grain boundaries with more thickness are indicated by white, red, yellow, blue, green, and dark arrows, respectively.

Fig. 4 depicts the OM and SEM microstructures of HR1 and HR2 hot-rolled samples with 84 % and 96 % reductions, respectively.

Hot-rolling in the temperature ranges of recrystallization and nucleation and growth of Laves phases causes a collision between recrystallization and precipitation. In this case, Laves phases usually prevent γ phase grain movement by locking the grain boundaries, and the grain size is controlled by them in the microstructure [33,34]. Researchers [31] demonstrated that, unlike the Laves phase, the oxide phase has little effect on grain boundary control and mostly intragranular precipitation. The pre-existing secondary-phase precipitates (Fig. 3a of the received sample) have a much lower ductility than the matrix, as seen in the HR1 sample microstructure (Fig. 4a). After hot-rolling, they are completely shredded, separated, and often randomly distributed inside the elongated grain. Also, considering the abundance of precipitates visible in sample HR1, in addition to the crushing of the precipitates of the previous secondary phases, due to the overlap of the hot-rolling temperature range with the formation range of secondary phases, there is the possibility of re-precipitation of these phases. According to the cases mentioned, the precipitates of the secondary-phases observed in Fig. 4a appear to be mostly the oxide phase particles with a non-uniform distribution in a non-recrystallized matrix (red arrows versus white arrows). Instead, the higher reduction in sample HR2 leads to more crushing of the remaining precipitates. Consequently, the size of the precipitates in this sample is very small (Fig. 4c yellow arrows). Due to the same heat treatment conditions as the HR1 sample, slight precipitation of the oxide phase is observed in the center of the HR2 sample compared to the HR1 sample. Also, the presence of annealing twins with no distortion (Fig. 4d blue arrows) and equiaxed grains in the microstructure of sample HR2 indicates that recrystallization occurred in this structure, as opposed to sample HR1 (Fig. 4b). The driving force required for the recrystallization phenomenon is the difference in misalignments in two adjacent grains caused by a difference in dislocation content on opposite sides of grain boundaries [35]. All these factors combine to create a duplex grain state and non-random cross-sectional conditions in the HR2 sample's microstructure after hot-rolling. As a result, the average grain size in this microstructure is about 17 μm at a distance of 200 μm near the surface and about 30 μm in the center. This duplex grain structure was referred to as a non-random microstructure with a local strain in ASTM E−1181 standard [36]. Appling local strains in the final passes of hot-rolling usually results in the gradual grain refinement of the microstructure. The grain size of the sample decreases as the distance from the center to the surface increases, indicating that gradual recrystallization occurs in each pass from the center to the surface. In addition, as the distance from the center to the surface of the sample increases, so does the thickness of the grain boundaries, with the average thickness ranging from 0.5 μm to 3 μm, as confirmed by the green arrows against the dark arrows in Fig. 4d. This indicates the presence of optimal conditions (sufficient temperature and time) for the precipitation of secondary-phases in newly recrystallized grain boundaries in the sample with less thickness. Previous studies [31,33] identified these particles as intergranular Laves phase based on the grain boundary of the precipitates of the secondary-phases observed in Fig. 4d.

Furthermore, the occurrence of this microstructure after thermomechanical treatment is predicted in AMS standards 5884, 5892, and 5893 [14]. Therefore, it appears that non-uniform and local (surface) strain causes an increase in recrystallized grains and the accumulation of intergranular Laves phase particles locally on the surface in the HR2 sample hot-rolled under 96 % reduction, resulting in surface refinement. On the other hand, the strain distribution is more uniform in sample HR1, which is hot-rolled under 84 % reduction, as a result of which sufficient stored energy will be provided for the recrystallization and nucleation of secondary-phases in the subsequent heat treatment throughout the structure.

**Fig. 5:**
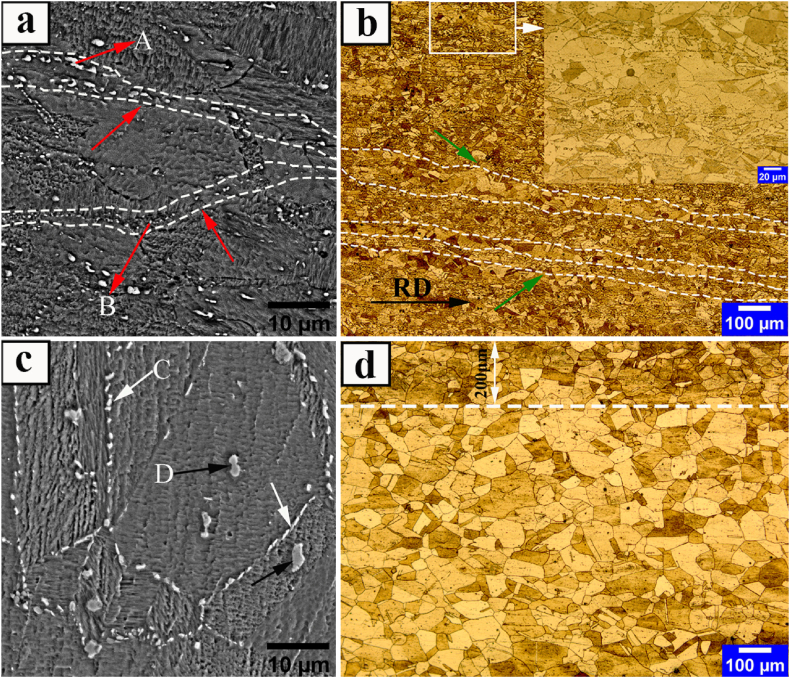
OM and SEM-BSE images of Incoloy 907 superalloy solution-treated samples; a-b) ST1 c-d) ST2. Secondary-phase particle bands, grain size bands, grain boundary Laves phase, and (Nb,Ti)O phase are represented by red, green, white, and black arrows, respectively.

Fig. 5 depicts the OM and SEM microstructures of ST1 and ST2 samples after solution-treatment at 980 °C, which were pre-hot-rolled at 84 % and 96 % reductions, respectively. A solution-treatment is required to dissolve the undesirable precipitates formed during the working process and controlled cooling. These undesirable precipitates limit the material's ability to form desirable precipitates during aging using strengthening alloy elements [1,2]. Excess Laves phase are also undesirable because lower residual niobium levels may reduce the precipitation of useful phases containing this element during aging [5]. Following the solution-treatment sample, ST1 images (Fig. 5a and b) show the simultaneous occurrence of static recrystallization and secondary-phase precipitation, resulting in a banded structure in the form of secondary-phase particle bands (red arrows) and grain size bands (green arrows).

Due to the high tendency of 900 series alloys to form a strong texture and anisotropic mechanical properties [[1], [2], [3]], secondary-phase particle bands and grain size bands are typically formed in the rolling direction in the microstructure. EDS analysis of several particles was used to investigate the chemical composition of the particles formed in the secondary-phase particle bands (Table 4). The analysis results in Table 4 show that the particles formed in the secondary-phase particle bands have a chemical composition that falls between the Laves and oxide phases.

**Table 4:** EDS analysis of the particles in the SEM microstructures of Fig. 5.

ELM (Wt%)	Fe	Ni	Co	Nb	Ti	Si	O
A	23.6	33.5	12.1	17.0	1.1	2.9	9.8
B	27.0	24.2	10.2	24.0	1.1	2.2	11.3
C	24.9	25.1	14.9	30.8	0.8	3.3	–
D	2.2	2.2	1.1	83.8	10.4	–	–

Due to recrystallization in the previous hot-rolling treatment in the ST2 sample, the solution-treatment dissolves Laves phase particles caused by the previous treatment and removes obstructions for the growth of surface and central grains. As a result, the effect of surface refinement has vanished in the ST2 sample, and the equiaxed grains with an average grain size of 48 μm are uniformly distributed throughout the microstructure (Fig. 5d). According to the chemical composition of the particles (Table 4) and the SEM image of the ST2 sample, the Laves phase is more visible in the grain boundaries (Fig. 5c white arrows), and the (Nb,Ti)O phase (Fig. 5c black arrows) is visible in the grain centers. The higher permeability coefficient of grain boundaries than grain centers, as well as the tendency to accumulate atoms of soluble and insoluble elements (such as niobium atoms segregation) at high temperatures, increase the likelihood of intergranular Laves phase precipitation [35,37,38]. Furthermore, a comparison of solution-treatment temperature (980 °C) with the dissolution temperature of Laves phase (950 °C) and (Nb,Ti)O phase (1175 °C) from the previous research [31] proves that most precipitates dissolved in the ST2 sample were of intergranular Laves phase.

**Fig. 6:**
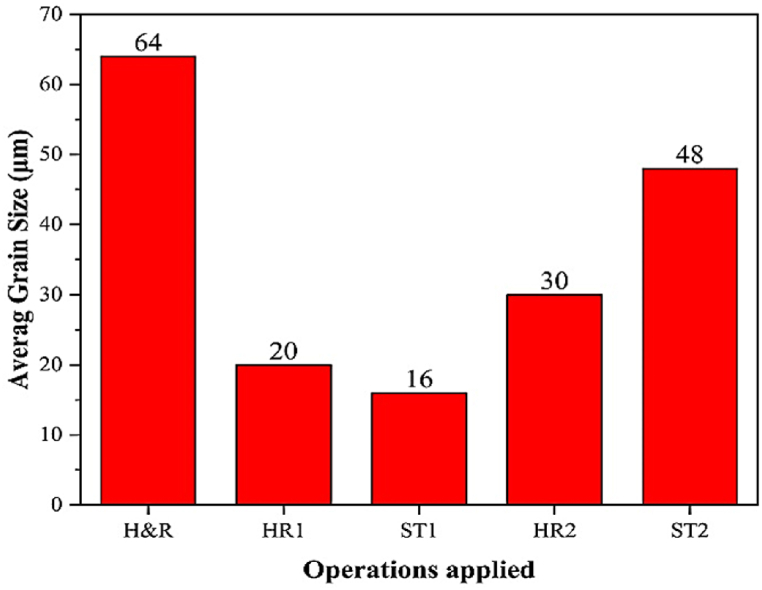
Average grain size of Incoloy 907 superalloy under various conditions.

The average grain size of Incoloy 907 alloy samples subjected to various hot-rolling and solution-treatments is shown in Fig. 6. As shown in Fig. 6, the average grain size decreases in both 84 % (HR1 and ST1 samples) and 96 % (HR2 and ST2 samples) reductions compared to the received sample (H&R). Furthermore, the average grain size of the samples under 84 % reduction shows a more decreasing trend than the samples under 96 % reduction. Therefore, the average grain size in 84 % reduction samples is in the 16–20 μm range and 30–48 μm in 96 % reduction samples. The reason for this is the recrystallization after hot-rolling in the HR2 sample, and the grain growth phenomenon is observed in the ST2 sample during the subsequent solution-treatment. Instead, after hot-rolling, the grains in the HR1 sample are elongated, and the recrystallization phenomenon occurs in the ST1 sample during the subsequent solution-treatment.

According to the Hall-Patch relationship [39,40], as the average grain size increases and the grain boundaries decrease, the dislocations’ movement increases and the strength decreases. Hence, a microstructure with a smaller average grain size achieves maximum strength and ductility. As a result of the lower average grain size, the hot-rolled samples under 84 % reduction with L\h_ave_ = 1.36, which were then subjected to solution-treatment, are expected to provide more favorable mechanical properties.

### 3.3 The effect of L/h_avg_ on the microstructure and tensile properties of aged samples

**Fig. 7:**
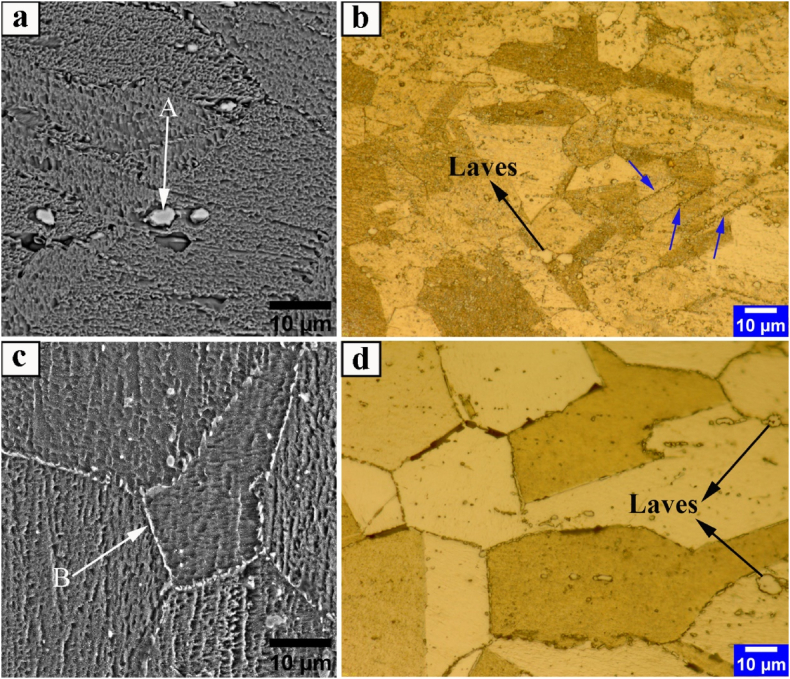
OM and SEM-BSE images of aged Incoloy 907 superalloy samples; a-b(DA1 c-d) DA2. Blue arrows indicate the retention of secondary-phase particle bands after aging.

Fig. 7 depicts the OM and SEM microstructures of DA1 and DA2 samples after aging, subjected to 84 % and 96 % reductions, pre-hot-rolled and pre-solution-treated, respectively. Table 5 also includes EDS analysis results of the secondary-phase particles observed in the microstructures of Fig. 7.

**Table 5:** EDS analysis of precipitates observed in SEM microstructures of Fig. 7.

ELM (Wt%)	Fe	Ni	Co	Nb	Ti	Si	O
A	1.1	0.9	0.56	87.0	10.2	–	–
B	33.4	29.9	13.1	20.2	1.5	0.5	1.3

According to the results, secondary-phase particle bands (Fig. 7b blue arrows) from the previous solution-treatment (ST1 sample) remained in the microstructure of the DA1 sample (Fig. 7b blue arrows). Also, the secondary-phase particles ((Nb, Ti)O, and Laves phases) in this sample are often observed in an intragranular form (Fig. 7a and b).

However, in the DA2 sample, the situation is slightly different. In this sample, unlike the previous solution-treatment (ST2 sample), the Laves phases have an intragranular morphology (Fig. 7d) in addition to the intergranular morphology (Fig. 7c). Due to the lower volume fraction of secondary-phase particles, sample DA2 has clearer grains than sample DA1, as shown in the OM images of Fig. 7.

**Fig. 8:**
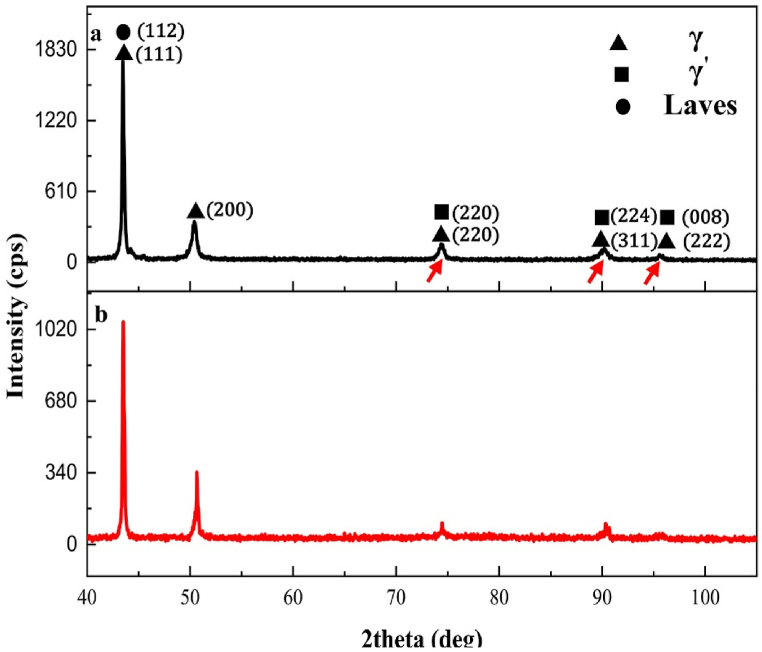
XRD results of Incoloy 907 superalloy aged samples: a) DA1 and b) DA2. Red arrows indicate γ'/γ phases peak.

Fig. 8 shows the XRD results of DA1 and DA2 samples to investigate finer-scale phases in the microstructure of aged samples. After the aging treatment in DA1 and DA2 samples, when the microstructure of Fig. 7 and the XRD results of Fig. 8 are combined, the ε phase was not precipitated. Moreover, the ε phase [(Ni, Co, Fe)_3_ (Ti, Nb)] has a hexagonal close-packed (HCP) structure and plate morphology. Thus, this phase immediately and directly precipitates from the supersaturated austenitic matrix in Incoloy 907 and Incoloy 909 superalloys when (1) temperature is below 770 °C, (2) time-duration is long with γ′ phase consumption, and (3) temperature is above 770 °C [41,42].

When the XRD analysis of the DA1 and DA2 samples are compared, the peaks associated with the γ'/γ phase (Fig. 8 of the red arrows) are more apparent and have a higher intensity in the DA1. The higher volume fraction of secondary-phase particles in the DA1 sample compared to the DA2 sample (Fig. 7b) and the XRD analysis results (Fig. 8a) indicate that the DA1 sample does not have the problem of reduced strengthing elements (such as titanium and niobium) for γ′ phase precipitation. This finding suggests that the aging hot-rolled sample at 84 % reduction (DA1 sample) improves γ′ phase precipitation.

**Fig. 9:**
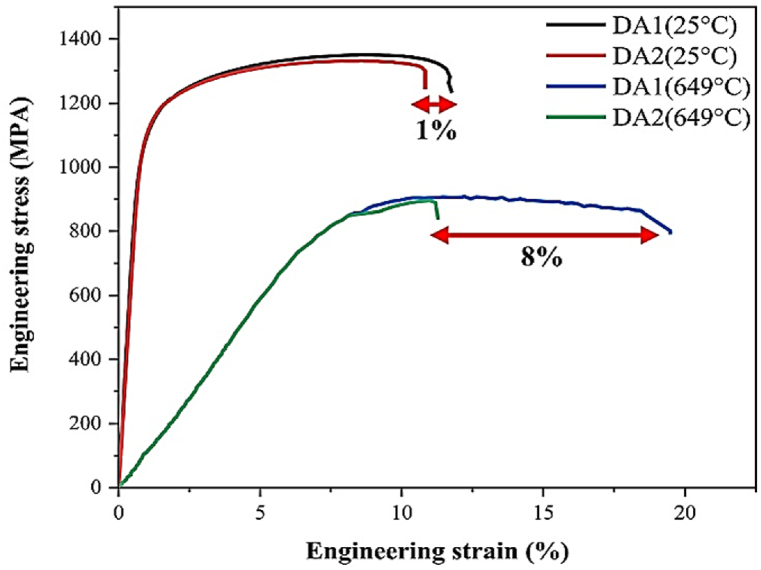
Engineering stress-strain diagram of aging DA1 and DA2 samples of Incoloy 907 superalloy at ambient and 649 °C temperatures.

The engineering stress-strain diagram of samples DA1 and DA2 tested at ambient and 649 °C temperatures is shown in Fig. 9. Since the Incoloy 907 superalloy's maximum working temperature is 649 °C, selecting 649 °C and the ambient temperature for the tensile test temperatures are practical. As shown in Fig. 9, sample DA1 has nearly the same strength (yield and tensile strength) as sample DA2 at ambient temperature and 649 °C. Furthermore, in dirty steels with numerous oxide phases elongated in the rolling direction, oxide phases do not affect yield strength [43].

Besides, Fig. 9 shows that the tensile test at 649 °C increases ductility compared to the ambient temperature. In general, increasing the temperature of the tensile test reduces strength while increasing ductility. At temperatures above 0.4 melting point, typical high-temperature deformation processes are controlled by glide dislocation and dynamic recovery. As a result, the yield strength decreases. Some factors can reduce the strength of superalloys at high temperatures, such as decreasing the strength of austenite solid solution and increasing the thermal energy required to cross the dislocations through the barriers [44].

On the other hand, sample DA1 at ambient and 649 °C temperatures exhibit a more extended elongation of about 1 % and 8 %, respectively, than sample DA2. This increase in ductility in the DA1 sample could be attributed to the banded structure created during the solution-treatment and preserved after aging (Fig. 7b, blue arrows). Tensile ductility is expected to decrease significantly in samples with oxide phase clusters in the form of lamination, such as DA1. However, the Laves phase, in addition to the (Nb, Ti)O phase in the banded structure of the DA1 sample, reverses this trend. Therefore, hot-rolled samples under 84 % reduction with L\h_ave_ = 1.36 in the aged state have maximum strength and hot and cold ductility.

## 4 Conclusion

The results of hot-rolling, solution, and aging treatments on Incoloy 907 superalloy, hot-rolled under of 84 % and 96 % reductions, respectively, show that:

1. The applied strain is uniform for hot-rolling under 84 % reduction, resulting in complete shredding of secondary-phase precipitates and their random distribution in the microstructure with elongated grains. In hot-rolling under 96 % reduction, however, local strain in successive passes, combined with the simultaneous occurrence of recrystallization and precipitation of secondary-phase particles, results in surface refinement and local accumulation of secondary-phase particles on the alloy's surface, which is referred to as a duplex grain, and non-random microstructures with different cross-sectional conditions.
2. Static recrystallization combined with the precipitation of secondary-phase particles in the pre-hot-rolled under 84 % reduction solution-treated sample results in forming banded structure in the form of grain size bands and secondary-phase particle bands with a chemical composition between the Laves phase and (Nb, Ti)O. Instead, after the solution-treatment, the dissolution of the Laves phase particles and grain growth in the hot-rolled sample under 96 % reduction resulted in eliminating structure duality and developing uniform equiaxed grains throughout the microstructure.
3. In the aged sample subjected to 84 % reduction, hot-rolled, and then solution-treated, compared to the aging sample subjected to 96 % reduction, hot-rolled, and then solution-treated, the γ'/γ phase is more apparent and intensified. This event increases cold and hot ductility by 1 % and 8 %, respectively, while maintaining the banded structure (secondary-phase particle bands) created by the previous solution-treatment.
4. In Incoloy 907 superalloy, L\h_ave_ = 1.36 is an optimal and normalized ratio for converting billet into sheet for direct use in structural applications since it results in a more appropriate microstructure and mechanical properties than L\h_ave_ = 2.87.

## Data availability statement

The authors do not have permission to share data.

## CRediT authorship contribution statement

**Seyed Sajjad Babaei Sangtabi:** Writing – original draft, Investigation, Conceptualization. **Seyed Mehdi Abbasi:** Writing – review & editing, Supervision, Methodology. **Rashid Mahdavi:** Methodology.

## Declaration of competing interest

The authors declare that they have no known competing financial interests or personal relationships that could have appeared to influence the work reported in this paper.
